# Unleashing viral mimicry: A combinatorial strategy to enhance the efficacy of PARP7 inhibitors

**DOI:** 10.1002/bies.202400087

**Published:** 2024-11-06

**Authors:** Patrick Manetsch, Michael O. Hottiger

**Affiliations:** ^1^ Department of Molecular Mechanisms of Disease University of Zurich Zurich Switzerland; ^2^ Molecular Life Science PhD Program of the Life Science Zurich Graduate School University of Zurich Zurich Switzerland

**Keywords:** anti‐tumor immunity, cancer therapy, FRA1, PARP7, programmed cell death, viral mimicry

## Abstract

Cancer cells exploit mechanisms to evade immune detection triggered by aberrant self‐nucleic acids (NA). PARP7, a key player in this immune evasion strategy, has emerged as a potential target for cancer therapy. PARP7 inhibitors reactivate NA sensing, resulting in type I interferon (IFN) signaling, programmed cell death, anti‐tumor immunity, and tumor regression. Cancer cells with elevated IFN‐stimulated gene (ISG) scores, representing a viral mimicry‐primed state, are particularly sensitive to PARP7 inhibition. This review focuses on the endogenous sources of NA in cancer and the potential to exploit elevated aberrant self‐NA in cancer therapy. We describe strategies to increase cytoplamic NA levels, including targeting epigenetic control, DNA damage response, and mitochondrial function. We also discuss targeting RNA processing pathways, such as splicing and RNA editing, to enhance the immunostimulatory potential of existing NA. Combining PARP7 inhibitors with NA elevating strategies may improve cancer immunotherapy, especially for tumors with high ISG scores.

## Graphical abstract

This review explores the potential of inducing a viral mimicry‐primed state by combining PARP7 inhibitors with drugs that promote the accumulation of self‐nucleic acids. This synergistic approach could enhance apoptosis and the immune response against cancer cells with elevated IFN‐stimulated gene (ISG) scores, providing a promising avenue for innovative treatment strategies. The graph was created with BioRender.com (license agreement number: MW27DFMCQI).

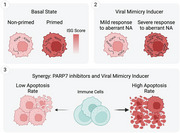

## INTRODUCTION

Tumor cells are adept at reprogramming their signaling pathways and altering their transcriptional, translational, and post‐translational machinery to overcome physiological and microenvironmental stresses.^[^ ^1^, ^2^ ^]^ This extraordinary ability of cancer cells to survive under stressful conditions is a major challenge in cancer therapy.^[^ ^3^ ^]^ Recently, it has been discovered that tumor cells often contain unusual amounts of and aberrantly located nucleic acids (NA). These NA serve as robust molecular signatures of perturbed cellular homeostasis.^[^ ^4^ ^]^ Endogenous or self‐DNA and ‐RNA play important roles in the innate immune response and can trigger cytosolic and endosomal pattern recognition receptors (PRRs), such as toll‐like receptors (TLRs) 3, 7, 8, and 9, absent in melanoma 2 (AIM2)‐like receptors (ALR),^[^ ^5^ ^]^ cyclic guanosine monophosphate‐adenosine‐monophosphate synthase (cGAS) ^[^ ^6^ ^]^ and retinoic acid‐inducible gene I (RIG‐I)‐like receptors (RLRs).^[^ ^7^ ^]^ Upon binding of NA to PRRs, the initiated downstream signaling cascade results in the activation of the transcription factors interferon regulatory factor 3 (IRF3), IRF7, Nuclear Factor‐kappa B (NF‐κB), and AP‐1, which promote the production of cytokines, including type I interferons (IFN). These cytokines subsequently orchestrate an innate and adaptive immune response, leading to tumor regression and durable immunity.^[^ ^8^, ^9^, ^10^ ^]^ Another consequence of PRR signaling is the induction of autonomous cell death programs that are triggered by multiple (i.e., apoptosis, pyroptosis, and necroptosis) and often overlapping (i.e., PANoptosis) pathways.^[^ ^4^, ^11^, ^12^ ^]^ To avoid cell death and/or recognition by the immune system, cancer cells rely on repressors of the NA‐induced innate immune response.^[^ ^13^, ^14^ ^]^ Based on these findings, it is not surprising that novel therapeutic agents leveraging the power of innate immunity induced by aberrant NA have gained considerable attention.^[^ ^15^ ^]^

PARP7 *(TIPARP)* has recently been identified as a potential anti‐cancer target due to its critical role in suppressing intra‐tumoral type IFN signaling.^[^ ^16^, ^17^, ^18^, ^19^ ^]^ PARP7 is a member of the ADP‐ribosyltransferase (ART) superfamily.^[^ ^20^ ^]^ Members of this superfamily transfer ADP‐ribose moieties from nicotinamide adenine dinucleotide (NAD^+^) ^[^ ^21^ ^]^ to substrates via N‐, O‐, or S‐glycosidic linkages on target proteins, resulting in the post‐translational modification ADP‐ribosylation.^[^ ^20^, ^22^, ^23^, ^24^, ^25^ ^]^ In humans, 21 proteins make up the ART superfamily.^[^ ^20^ ^]^ This superfamily is based on the homology of their catalytic domain with bacterial toxins and is thus further subdivided into two enzyme families: the cholera toxin‐like ARTs (ARTCs) and the diphtheria toxin‐like ARTs (ARTDs).^[^ ^26^ ^]^ The ARTD family represents the larger subset of ARTs and consists of 17 enzymes that catalyze either mono‐ or poly‐ADP‐ribosylation, depending on the composition of their catalytic domain.^[^ ^20^ ^]^ While PARP7 catalyzes mono‐ADP‐ribosylation (MARylation) on cysteine residues, the most studied member of this enzyme family, PARP1, a key sensor in the DNA damage response, primarily generates serine‐linked poly‐ADP‐ribosylation (PARylation).^[^ ^19^, ^24^, ^27^ ^]^

**FIGURE 1:**
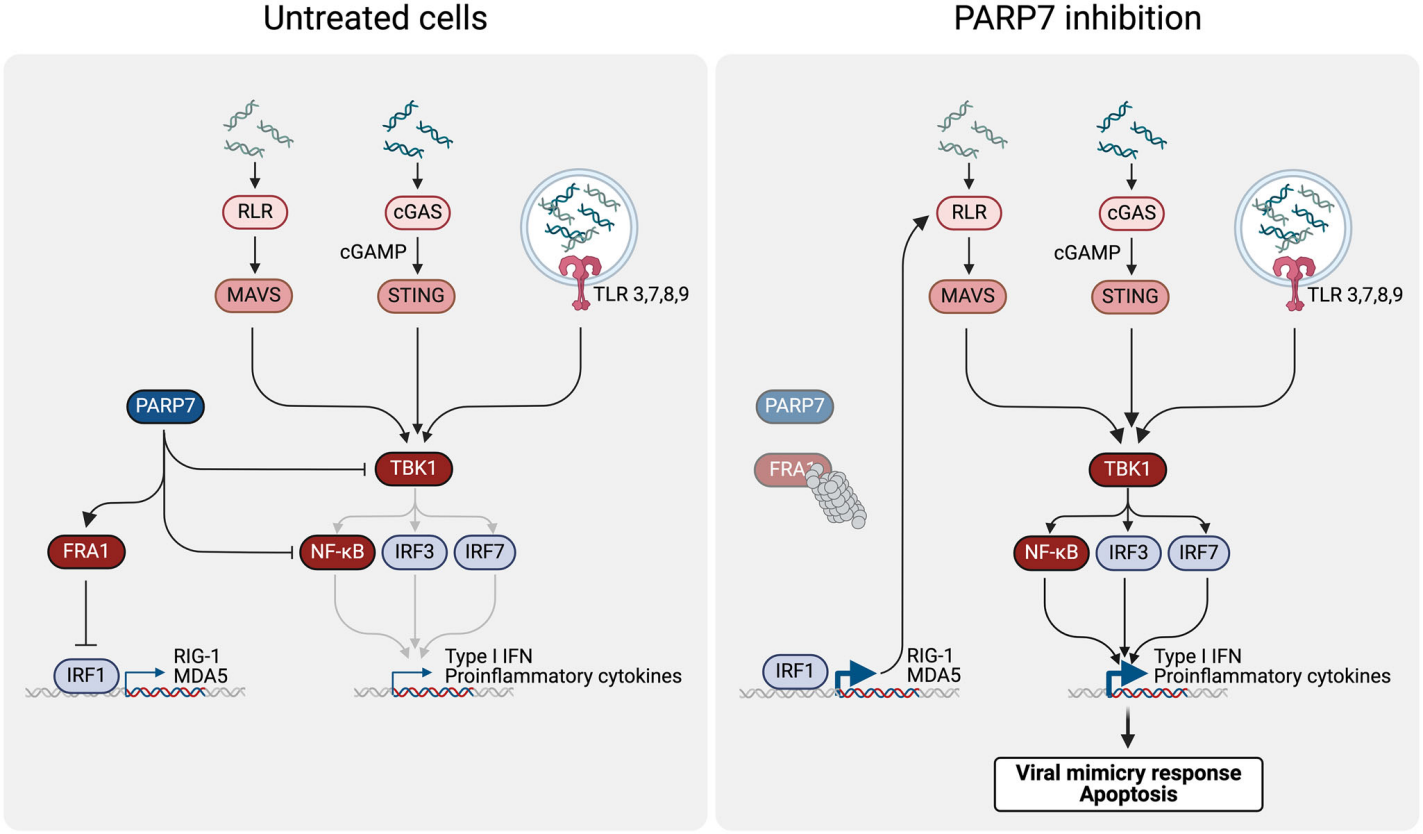
PARP7 inhibition unleashes type I IFN signaling and promotes apoptosis by regulating FRA1, TBK1, and NK‐κB activity. In PARP7 inhibitor‐sensitive cancer cells, PARP7 stabilizes FRA1 and represses TBK1 and RELA in a MARylation‐dependent manner. Thus, PARP7 activity suppresses apoptosis and an innate immune response triggered by cytosolic and endosomal self‐DNA and ‐RNA. Upon PARP7 inhibition, the proteasomal degradation of FRA1 and the activation of TBK1 and RELA unleashes type I IFN signaling and promotes apoptosis. The graph was created with BioRender.com (license agreement number: CZ26MOZM4K).

In tumor cells, PARP7 inhibition using PARP7‐selective small molecules^[^ ^16^, ^28^, ^29^, ^30^ ^]^ restored cytoplasmic NA‐dependent type I IFN signaling and reduced cancer cell growth in a cell‐autonomous manner (Figure 1). PARP7 inhibition also contributed to tumor regression by enhancing immune recognition of cancer cells, thereby establishing a durable state of immunity.^[^ ^16^ ^]^ While the mode of action of PARP7 inhibitors has been mainly studied in cancer cells, it was recently discovered that PARP7 inhibition also enhances the anti‐tumor activity of immune cells, including pro‐inflammatory macrophages and CD8^+^ T cells.^[^ ^18^ ^]^ At a molecular level, PARP7 was initially proposed to promote cancer cell viability and suppress intra‐tumoral and immune cell cytokine expression by modifying and inhibiting TANK‐binding kinase 1 (TBK1) (Figure 1).^[^ ^16^, ^31^ ^]^ However, recent evidence suggests that PARP7 inhibition modulates immune signaling and cell survival pathways, independently of its effects on TBK1 activity.^[^ ^17^, ^18^ ^]^ Recently, in murine breast carcinoma cells, PARP7 has been suggested to interact with and modify RELA (Figure 1), a key subunit of NF‐κB.^[^ ^18^ ^]^ However, there is currently no further evidence that PARP7 directly affects RELA phosphorylation and activity, especially in human cancer cells. Furthermore, the question of whether RELA is modified by PARP7 and the functional implications of such modification requires further investigation. Finally, our group has recently identified a subset of PARP7‐dependent cancer cells that are driven by the AP1 transcription factor and oncogene FRA1 *(FOSL1)*. In cancer cells expressing elevated levels of PARP7 and FRA1, PARP7 inhibition resulted in the proteasomal degradation of FRA1. Loss of FRA1 promoted an IRF1‐ and IRF3‐dependent induction of type I IFN signaling via the upregulation of the RLR RIG‐1 and melanoma differentiation‐associated protein 5 (MDA5), as well as CASP8‐mediated apoptosis (Figure 1).^[^ ^19^ ^]^ Taken together, these findings suggest that the PARP7 inhibition‐dependent activation of IRF3, IRF7, and NK‐κB inhibits tumor growth (Figure 1). Thus, the amount and nature of self‐DNA or ‐RNA may determine the efficacy of PARP7 inhibitors. Indeed, co‐treatment of cancer cells with PARP7 inhibitors and PRR agonists synergistically induced type I IFN signaling and apoptosis.^[^ ^16^, ^17^, ^19^ ^]^

The mechanisms driving the accumulation of aberrant NA species in cancer cells have only recently been investigated. Understanding the systems that drive the intracellular accumulation of self‐DNA and ‐RNA is important because their induction could potentially be exploited therapeutically in combination with the PARP7 inhibitor RBN‐2397, which is being evaluated in clinical trials (NCT04053673). While RBN‐2397 has shown promising results in clinical trials,^[^ ^32^ ^]^ its specificity to PARP7 remains a subject of debate. In PARP7 knockout cells, the compound retained anti‐tumor activity, suggesting potential off‐target effects on other ARTs such as PARP2.^[^ ^16^ ^]^ To overcome these limitations and to gain a clearer understanding of the role of PARP7 in tumor growth, researchers have developed more potent and selective PARP7 inhibitors.^[^ ^17^, ^29^, ^30^ ^]^ Compounds such as KMR‐206^[^ ^17^ ^]^ not only refined the targeting of PARP7, but also confirmed its anti‐tumor mechanism, involving NA‐dependent immune signaling and apoptosis.^[^ ^17^, ^29^, ^30^ ^]^ Despite differences in potency and specificity among available PARP7 inhibitors, their underlying mechanism is largely consistent. Therefore, in this review, we will refer to these compounds collectively as PARP7i and focus on the broader consequences of PARP7 inhibition. In addition, to its use as a monotherapy, the anti‐tumor activity of PARP7i is currently being investigated in combination with the immune checkpoint inhibitor (ICI) pembrolizumab, an anti‐PD1 monoclonal antibody (NCT05127590). There is growing evidence suggesting that the combinatorial use of ICIs with therapeutic agents that enhance NA‐mediated immunity may provide new strategies for tumor control.^[^ ^15^ ^]^ Therefore, enhancing the response to PARP7i by interventions that increase the levels of aberrant NA may further promote synergy with ICIs. In this review, we examine the molecular mechanisms that govern the levels of self‐NA in cancer cells and the treatments that increase abnormally localized DNA and RNA. Moreover, we highlight how these treatments may work in concert with PARP7i to induce type I IFN signaling and apoptosis. Our discussion focuses on cancer cells that have previously been characterized as being primed for viral mimicry induction, as they are particularly susceptible to accumulating NA.

## PARP7‐ and FRA1‐dependent cell lines are in a viral mimicry‐primed state

**FIGURE 2:**
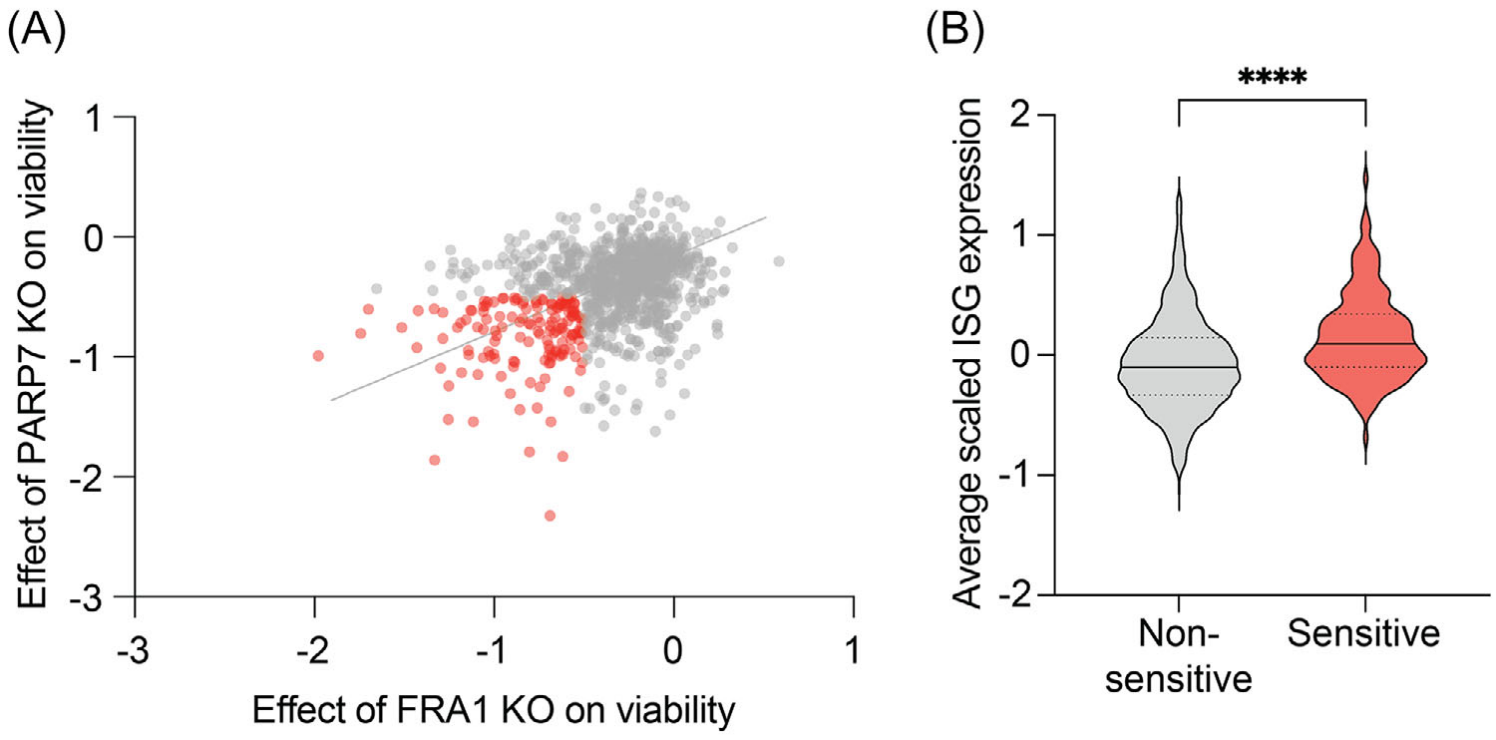
Elevated ISG score in PARP7‐ and FRA1‐dependent cell lines indicates a viral mimicry‐primed state. (A) Linear correlation between the effect of PARP7 and FRA1 knockout on cell viability from the CRISPR knockout dataset from the Dependency Map Portal (DepMap, release: Public 24Q2, *n* = 1021). The CERES score measures the size of the fitness difference that is observed in a pooled screen from a single gene in a single mammalian cell line. Here, the score represents the effect size of PARP7 or FRA1 knockout on cell viability, normalized against a distribution of non‐essential and pan‐essential genes. Red data points indicate cell lines that are dependent on PARP7 and FRA1 using the CERES cut‐off of ≤ −0.5. Spearman correlation = −0.33, *p*‐value for linear correlation = 1.6 × 10^−28^. (B) Comparison of the average scaled expression of ISGs from the DepMap Portal (release: Public 24Q2, *n* = 1021) between non‐sensitive and PARP7‐ and FRA1‐sensitive cell lines. The ISG score is presented as the mean of Z‐score transformed log(TPM+1) values of 38 ISGs.^[^ ^33^ ^]^ Significance was determined by Student's *t*‐test **** *p* < 0.0001.

A major challenge in sensitizing cancer cells to PARP7 inhibition by increasing the levels of self‐DNA or ‐RNA is to identify cell lines that will respond to the aberrant accumulation of double‐stranded NA. A recent report suggested that cell lines with elevated baseline expression of 38 IFN‐inducible genes (ISG) are more susceptible to respond to cytosolic double‐stranded (ds)RNA due to the upregulation of the RLR RIG‐1, MDA5, and protein kinase R (PKR).^[^ ^33^ ^]^ The initial and basal increase in ISG expression was found to be induced by cytosolic DNA triggering the cGAS stimulator of interferon genes (STING) pathway.^[^ ^33^ ^]^ Therefore, the authors suggested that cancer cells with pre‐existing high levels of self‐DNA might be more susceptible to the effects of additional cytosolic dsRNA.^[^ ^33^ ^]^ These findings highlight that a subset of tumor cells with elevated ISG scores are primed for a viral mimicry response.^[^ ^34^ ^]^ A viral mimicry‐primed state describes a cellular state of active antiviral response triggered by endogenous NA.^[^ ^34^ ^]^ Moreover, these cells may be more susceptible to treatments that result in the accumulation of aberrant NA. A growing body of evidence suggests that cancer cells with elevated ISG expression are under constant selective pressure to suppress a potentially deleterious innate immune response induced by aberrant NA. Indeed, cells that are primed for viral mimicry have been found to depend for their survival on the repressors of dsRNA, including adenosine deaminase acting on RNA (ADAR1) and 5′‐3′‐ exoribonuclease 1 (XRN1).^[^ ^33^, ^34^, ^35^, ^36^ ^]^ Taken together, these findings suggest that cancer cells that are in a viral mimicry‐primed state may be more sensitive to interventions that disrupt the mechanisms that prevent the accumulation of self‐DNA and ‐RNA and, thus, may be more susceptible to a potential synergy with PARP7i. In support of this idea, PARP7‐ and FRA1‐dependent cell lines (Figure 2A) in the Dependency Map dataset (DepMap, https://depmap.org/portal/) show a significantly increased ISG score (Figure 2B). Furthermore, we found that the dsRNA‐dependent IRF3 activation was most critical for the induction of apoptosis in PARP7‐ and FRA1‐dependent cancer cells.^[^ ^19^ ^]^ Therefore, these findings suggest a direct link between the levels of aberrant NA and IRF3 activation, which in turn drives apoptosis. This vulnerability could be exploited in FRA1‐driven and PARP7i‐sensitive cancer cells by treatments that increase NA levels. We will discuss the dysregulated systems that contribute to NA accumulation and potential therapeutic strategies to further increase NA levels and enhance the response to PARP7i in these cancer cells.

## ENDOGENOUS SOURCES OF ABERRANT CYTOSOLIC AND ENDOSOMAL NA IN CANCER CELLS

**FIGURE 3:**
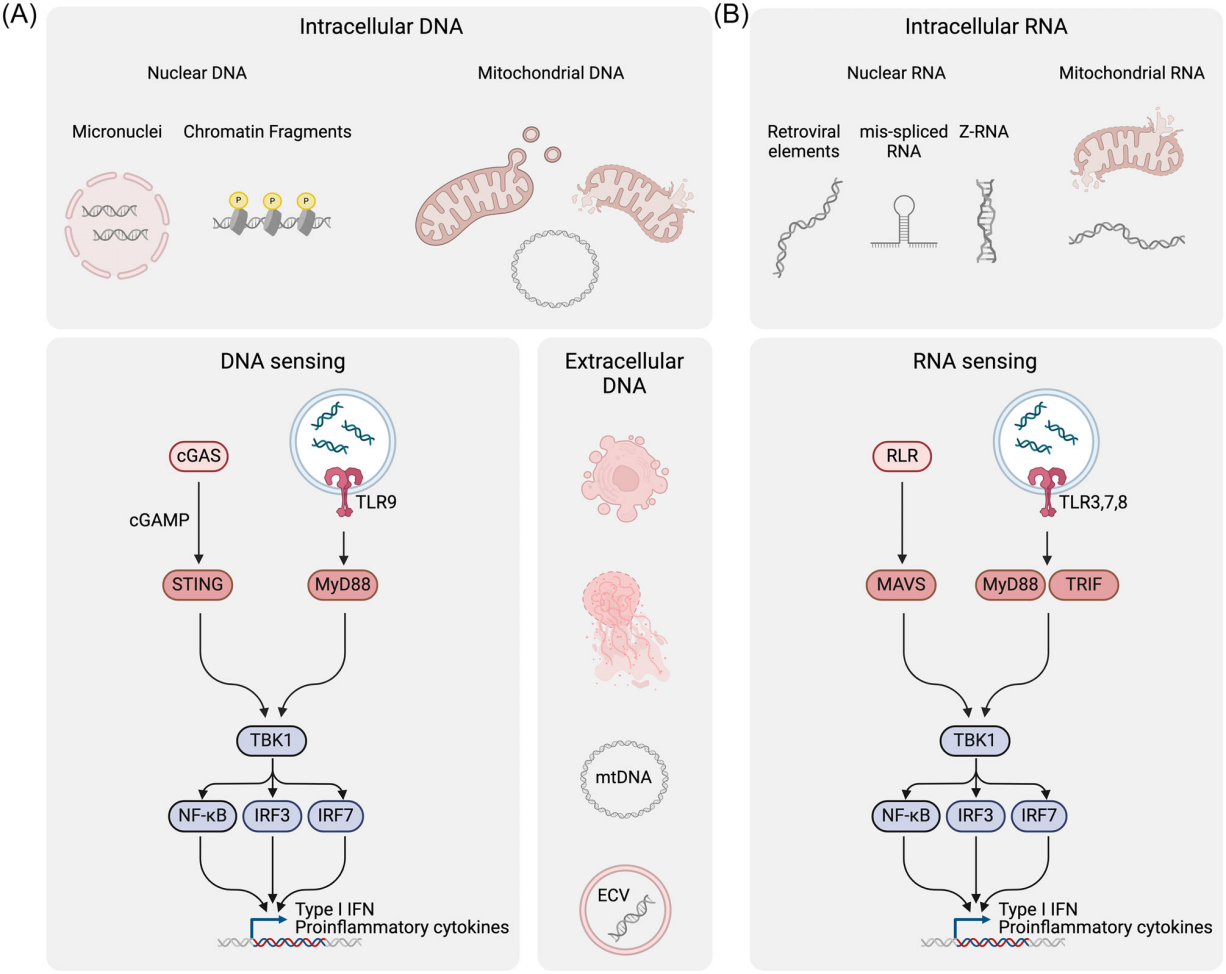
Aberrant NAs trigger innate immune signaling: intracellular vs. extracellular sources. (A) DNA‐dependent innate immune signaling can be initiated by (1) intracellular DNA originating from nuclear or mitochondrial sources or by (2) internalized extracellular gDNA and mtDNA from dying and vesicle‐producing cells as well as NETs from neutrophils. Cytosolic or endosomal abnormal self‐DNA triggers the cGAS‐STING or TLR9 pathway, respectively. (B) mtRNA and nuclear encoded RNAs including retroviral elements, mis‐spliced mRNAs and Z‐RNAs can activate cytosolic or endosomal RNA sensing via the RLR‐MAVS pathway or via TLR3, TLR7, and TLR8. The graph was created with BioRender.com (license agreement number: DP26MP58UU).

PARP7‐ and FRA1‐dependent cell lines exhibit an elevated ISG score (Figure 2A,B), indicating that they have intrinsic defects that prime them for viral mimicry through abnormal self‐DNA and ‐RNA. Aberrant NA can originate from multiple extracellular and intracellular sources (Figure 3A,B).^[^ ^37^ ^]^ Intracellular NA represents the largest pool of self‐NA and includes genomic DNA (gDNA), mitochondrial DNA (mtDNA), and nuclear or mitochondrial RNA. While membranes segregate gDNA and mtDNA from intracellular DNA sensors, post‐transcriptional modifications of endogenous RNA limit its ability to stimulate cytosolic RNA sensors.^[^ ^38^ ^]^ One of the main principles of NA sensing is to distinguish self from non‐self and altered‐self NA. To reliably discriminate altered‐self NA, NA sensors integrate information about the structure, the availability, and the localization.^[^ ^38^ ^]^ The following section will review the primary sources, forms, and properties of endogenous NAs and how they activate their respective NA sensors.

### Endosomal and cytosolic DNA sensing

gDNA can accumulate in the cytosol through the rupture of micronuclei (MN) or as chromatin fragments (Figure 3A).^[^ ^39^, ^40^ ^]^ MN consist of whole chromosomes or chromosome fragments separated from the cytoplasm by a membrane. MN formation occurs during cell division and is associated with whole chromosome or chromosome fragment mis‐segregation events induced by mitotic defects or DNA mis‐repair, respectively.^[^ ^41^ ^]^ The integrity of the MN nuclear envelope can be spontaneously lost, thus leading to rupture and recognition of MN DNA by cytosolic DNA sensors.^[^ ^42^ ^]^ In addition, gDNA can also be present in the cytosol as chromatin fragments that are highly enriched for the DNA damage mark γH2AX, further suggesting that DNA damage plays an important role in their formation.^[^ ^43^ ^]^ In addition to the nucleus, cytosolic DNA can also originate from mitochondria (Figure 3A).^[^ ^44^ ^]^ Recent reports suggest that loss of mitochondrial integrity and compartmentalization due to mitochondrial stress could lead to cytosolic release of mtDNA.^[^ ^45^ ^]^ These mitochondrial stressors include the excessive accumulation of reactive oxygen species (ROS), sublethal and lethal DNA damage, and the accumulation of fumarate.^[^ ^46^, ^47^, ^48^ ^]^ mtDNA can be released through two types of mitochondrial outer membrane (MOM) pores: (1) either through widespread or limited MOM permeabilization (MOMP) events mediated by BAX/BAK, or (2) through the Ca^2+^ and VDAC1‐dependent formation of the mitochondrial permeability transition pore (mPTP).^[^ ^46^, ^48^ ^]^ The ROS‐dependent release of mtDNA renders it more susceptible to oxidation, a modification that makes mtDNA more resistant to nuclease‐mediated degradation and enhances its immunostimulatory potential.^[^ ^49^ ^]^ The release of mtDNA is not limited to MOMP and mPTP events but can also be selectively mediated by mitochondria‐derived vesicles.^[^ ^50^ ^]^ In addition to intracellular gDNA and mtDNA, cell‐free extracellular DNA (cfDNA) has been found to activate type I IFN signaling directly at the cell surface or through endosomal and cytosolic DNA sensors following the uptake via endocytic pathways (Figure 3A).^[^ ^51^ ^]^ Extracellular cfDNA is mainly derived from apoptotic and necrotic hemopoietic cells and is characterized by a short (<200 bp) or long length, depending on the mode of cell death.^[^ ^51^, ^52^ ^]^ Moreover, neutrophil extracellular traps (NET) released by activated neutrophils during NETosis can promote innate immune signaling.^[^ ^52^ ^]^ Given the high turnover rate of tumor cells, it is not surprising that serum cfDNA levels increase with tumor growth due to ineffective clearance of dead and dying tumor cells.^[^ ^53^ ^]^ The elevated serum cfDNA levels can be utilized for liquid biopsies and aid in the early detection of tumorigenesis.^[^ ^54^ ^]^

The cGAS‐STING pathway is the primary cytosolic DNA sensor responsible for the induction of type I IFN signaling (Figure 3A).^[^ ^13^ ^]^ Several other receptors have also been implicated in the immune recognition of cytosolic DNA, including DHX9, DHX36, DDX41, ZBP1, MRE11, DNA‐PK, XRCC6, XRCC5, PYHIN1, AIM2, and IFI16.^[^ ^38^ ^]^ Notably, all except DHX9 and DHX36 appear to function upstream of STING.^[^ ^38^ ^]^ This abundance of DNA sensors suggests potential functional redundancy or cell type‐specific roles. However, the precise molecular mechanisms of these overlapping cytosolic DNA sensing pathways remain elusive. Given the synergistic type I IFN induction observed in PARP7‐dependent cancer cells treated with RBN‐2397 and a STING agonist,^[^ ^16^, ^28^ ^]^ we will focus here on the cGAS‐STING pathway. Cytosolic dsDNA recognition by cGAS triggers the generation of the second messenger, 2′3'‐cGAMP. This ligand binding induces a conformational change in STING, promoting its oligomerization into tetramers and higher‐order structures. Subsequently, these STING complexes dissociate from ER anchor proteins and are encapsulated in COPII vesicles for transport to the Golgi apparatus. At the Golgi, STING oligomers recruit and activate TBK1, leading to its autophosphorylation and subsequent phosphorylation of STING itself. Activated STING serves as a docking platform for IRF3, facilitating its phosphorylation by TBK1. Dimerized and phosphorylated IRF3 translocates to the nucleus to initiate the transcription of type I IFNs and the subsequent expression of a diverse array of ISGs. Notably, STING additionally stimulates NF‐κB activity through TBK1‐dependent and ‐independent mechanisms, culminating in the IκBα phosphorylation and nuclear translocation of NF‐κB, ultimately promoting the production of inflammatory cytokines.^[^ ^13^, ^55^, ^56^, ^57^, ^58^ ^]^ In addition to its canonical function and localization in the cytosol, cGAS has been shown to be tightly bound to nucleosomes and to play a role in DNA damage repair.^[^ ^59^, ^60^ ^]^ Furthermore, its ability to sense cytosolic DNA was found to be dependent on the DNA damage response, during which MRE1111 releases cGAS from nucleosomes.^[^ ^61^ ^]^ PARP7 inhibition also promotes the anti‐tumorigenic effect of immune cells, suggesting a central role for NA sensing in immune cell function.^[^ ^18^ ^]^ Indeed, the uptake of tumor‐derived DNA by dendritic cells (DCs) can activate the cGAS‐STING pathway, leading to type I IFN production and the subsequent cross‐priming of CD8^+^ T cells for anti‐tumor immunity.^[^ ^62^ ^]^ While the cGAS‐STING pathway is essential for responding to aberrant cytosolic DNA, TLR9 is the only known endosomal DNA sensor (Figure 3A).^[^ ^63^ ^]^ TLR9 preferentially recognizes single‐stranded (ss) DNA containing unmethylated cytosine‐phosphate‐guanine (CpG) motifs, which are less abundant in eukaryotic self‐gDNA compared to mitochondrial and bacterial DNA.^[^ ^64^ ^]^ Internalization of extracellular mtDNA or endogenous mtDNA by endocytic or autophagic pathways, respectively, can trigger TLR9‐mediated innate immune responses via IRF7 and NF‐κB.^[^ ^65^ ^]^ Finally, the pool of aberrant cytosolic DNA and RNA are interrelated, as cytosolic DNA can be transcribed by RNA polymerase III, leading to the activation of the RLR RIG‐1.^[^ ^66^, ^67^ ^]^ Similarly, the translocation of transposable elements can trigger the cGAS‐STING pathway through the reverse transcription of RNA into double‐stranded DNA.^[^ ^68^ ^]^

### Endosomal and cytosolic RNA sensing

Endogenous RNAs are poor stimulators of endosomal and cytosolic RNA sensors due to their post‐transcriptional modifications and biochemical properties.^[^ ^37^ ^]^ Nevertheless, actively transcribed and, thus, reawakened endogenous retroviral elements (EREs), which constitute a large portion of the human genome and are capable of forming dsRNA structures, have emerged as a potent means of stimulating innate immune signaling (Figure 3B).^[^ ^69^ ^]^ EREs can be transcribed from LTR‐containing endogenous retroviruses (ERV) or from non‐LTR repeat sequences such as long or short interspersed nuclear elements (LINE/SINE).^[^ ^70^, ^71^ ^]^ The reawakening of ERE‐derived dsRNA is often accompanied by cancer‐associated epigenetic reprogramming resulting from widespread loss of DNA methylation or heterochromatin.^[^ ^72^ ^]^ In addition to epigenetic alterations, tumor transcriptomes show evidence of deregulated RNA splicing, including aberrant intron retention and alterations in both canonical and alternative splicing (Figure 3B).^[^ ^73^ ^]^ Oncogenic alterations, such as the hyperactivation of transcription factors like Myc and FRA1, burden the spliceosome and increase the reliance on pre‐mRNA splicing components.^[^ ^73^ ^]^ Dysregulation of the spliceosome in cancer cells can lead to the cytoplasmic accumulation of mis‐spliced mRNAs, many of which form dsRNA or circular structures.^[^ ^74^ ^]^ These endogenous dsRNAs are recognized by dsRNA‐binding proteins, triggering antiviral signaling and extrinsic apoptosis.^[^ ^73^, ^75^ ^]^ In addition, several PTMs have been identified that play a role in the structural rearrangement and spatial distribution of ribonucleoprotein particles, in particular the hnRNP and snRNP families, as well as spliceosome‐associated factors.^[^ ^76^ ^]^ Notably, PARP7 has been found to modify several splicing regulators and to promote the oncogenic activity of FRA1, suggesting that PARP7 may contribute to dsRNA accumulation by disrupting normal spliceosome function.^[^ ^19^, ^24^, ^77^ ^]^ Moreover, there is increasing evidence that mitochondrial RNAs (mtRNA), released during mitochondrial dysfunction, can activate the innate immune response (Figure 3B).^[^ ^47^, ^48^, ^78^, ^79^ ^]^ mtRNA is generated by the bidirectional transcription of the circular mitochondrial genome, resulting in long complementary RNAs.^[^ ^80^ ^]^ Based on its structural features, mtRNA has long been thought to preferentially trigger type I IFN signaling via MDA5.^[^ ^48^ ^]^ However, recent reports suggest that mtRNA also drives cytosolic RNA sensing by activating RIG‐1 and PKR.^[^ ^47^, ^79^ ^]^ While endogenous dsRNA typically adopts an A‐RNA conformation, some self‐RNAs have been shown to be prone to adopting a higher‐energy and left‐handed Z‐RNA conformation (Figure 3B).^[^ ^81^ ^]^ These RNAs are potent activators of MDA5 and ZBP1 and can mediate an innate immune response and necroptosis.^[^ ^82^, ^83^ ^]^ Surprisingly, the Z‐RNA confirmation was found to be overrepresented in the 3′ UTR of ISG mRNAs.^[^ ^81^ ^]^ This suggests that in PARP7‐ and FRA1‐dependent cancer cells, the basal expression of ISGs may form a positive feedback loop that further sensitizes ISG‐positive cells to respond to inappropriate cytoplasmic NA accumulation. Finally, there is increasing evidence suggesting that cells may accumulate immunostimulatory dsRNAs when there is a defect in RNA processing and degradation.^[^ ^84^ ^]^ Therefore, deficiencies in RNA metabolic enzymes, such as Dicer, XRN1 and SKIV2L are linked to the accumulation of dsRNA and type I IFN signaling and might prime cancer cells for a viral mimicry state.^[^ ^35^, ^36^, ^85^ ^]^

In the cytosol, abnormal dsRNA can be sensed by the RLR (Figure 3B), including RIG‐1, MDA5, LGP2, PKR, and ZBP1.^[^ ^14^, ^86^, ^87^ ^]^ In addition, members of the DExD/H‐box RNA helicases, the IFIT, and the OAS families, the ARTs PARP9, PARP13, and PARP14 as well as NLRP1 are involved in the response to self and foreign dsRNA.^[^ ^88^, ^89^, ^90^, ^91^ ^]^ Activation of the RLR pathway occurs when RNA ligands trigger RIG‐I and MDA5, leading to homotypic interaction of their CARD domains with a CARD domain on the adaptor molecule mitochondrial antiviral signaling protein (MAVS). This, in turn, activates MAVS, causing it to oligomerize on mitochondria and peroxisomes and triggering a downstream signaling cascade via TBK1 that activates IRF3, IRF7, and NF‐κB. LGP2 lacks the CARD domain and is therefore unable to activate MAVS. However, it interacts with MDA5 and RIG‐I to promote and regulate their functions.^[^ ^14^, ^86^ ^]^ RIG‐I and MDA5 recognize 5′‐triphosphate(ppp)‐containing panhandle dsRNA and long dsRNA, respectively.^[^ ^85^ ^]^ In addition to MDA5, long dsRNA can also activate PKR, which not only triggers a type I IFN response,^[^ ^35^ ^]^ but also contributes to the global translational shutdown by inhibiting eIF2α and cell death.^[^ ^33^, ^92^, ^93^ ^]^ Moreover, there is increasing evidence indicating that the Z‐RNA sensor ZBP1 plays a crucial role in innate immune or inflammatory signaling, as well as different forms of cell death.^[^ ^81^, ^86^ ^]^ Recently, ZBP1 was found to be a central signaling hub for the induction of PANoptosis, which includes the superimposed activation of apoptosis, necroptosis, and pyroptosis.^[^ ^11^, ^12^, ^94^ ^]^ In addition to the cytosol, ssRNA and dsRNA can induce a type I IFN response in endosomes.^[^ ^37^ ^]^ While TLR3 recognizes dsRNA and signals via TRIF, TLR7, and TLR8 bind to ssRNA and activate their downstream effectors via Myd88 (Figure 3B).^[^ ^8^, ^95^ ^]^ In contrast to DNA, which can be rapidly detected once aberrantly localized, RNA editing enzymes in the nucleus and the cytosol are essential for limiting the immunostimulatory potential of endogenous RNAs.^[^ ^96^ ^]^ Recent research has uncovered the role of ADAR1 as a critical repressor of immune responses activated by various sources of self‐RNA, regulated in part by the introduction of adenosine to inosine edits in their sequences.^[^ ^81^, ^97^, ^98^, ^99^, ^100^ ^]^ In addition to introducing A to I edits, ADAR1 can bind and mask the canonical A form of dsRNA, as well as Z‐RNA, through its IFN‐inducible p150 isoform.^[^ ^81^ ^]^ ADAR1 is essential for cellular and tissue homeostasis by regulating the onset of autoimmune diseases and by suppressing anti‐tumor immunity and senescence.^[^ ^81^, ^97^, ^98^, ^99^, ^100^ ^]^ In addition to inosine, N^6^‐Methyladenosine (m6A) is among the most abundant internal modification on RNAs. Compelling evidence suggests a crucial role of m6A modification in type I IFN signaling by regulating the immune stimulatory potential and secondary structure of RNAs.^[^ ^101^ ^]^

## VIRAL MIMICRY INDUCERS

**FIGURE 4:**
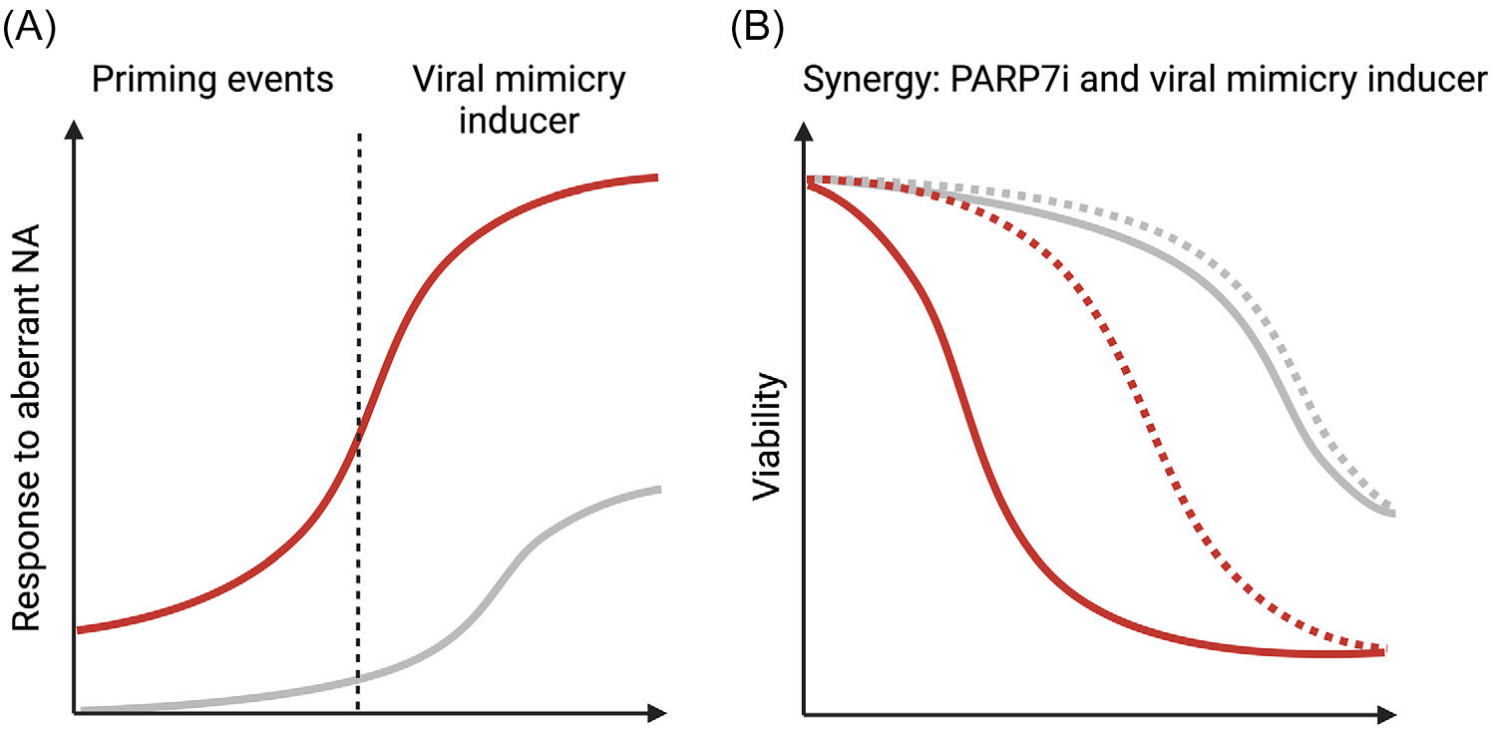
Inducers of viral mimicry potentially synergize with PARP7 inhibitors to promote apoptosis in FRA1‐driven tumors. (A) Tumorigenesis can be attributed to various factors such as mutations, epigenetic reprogramming, oncogene alteration, or mitochondrial dysfunction. These factors are present in FRA1‐ and PARP7‐dependent cell lines (red line) and act as priming events that elevate aberrant NA levels, which in turn increase their susceptibility to booster events that further derepress NA safeguards in comparison to non‐sensitive cells (grey line). (B) The increased susceptibility of cancer cells to accumulating NA suggests that the inhibition of PARP7 (PARP7i, dashed line) in combination with boosters of viral mimicry (solid line) may have a synergistic effect on the induction of programmed cell death. The red and grey line indicate FRA1‐ and PARP7‐dependent and non‐sensitive cell lines, respectively. The graph was created with BioRender.com (license agreement number: XU26MP4BY7).

Although elevated basal levels of NA can prime cells for a viral mimicry‐like state, baseline levels of IFNs and ISGs are not reliable predictors of the potential to enhance tumor immunogenicity. This is primarily because cancers can evade type I IFN signaling to tolerate elevated NA levels.^[^ ^72^ ^]^ Therefore, treatments that further elevate NA levels may provide an opportunity to promote anti‐tumor immunity and programmed cell death via viral mimicry (Figure 4A,B). This may be particularly true for FRA1‐positive and PARP7i‐sensitive cell lines with elevated ISGs scores (Figure 2A,B), as they represent a niche of cancer cells that are poised to respond to the accumulation of aberrant NA.^[^ ^33^ ^]^ Therefore, the combination of viral mimicry inducers and PARP7i may provide new opportunities for tumor control, especially in combination with ICI (Figure 4A,B).^[^ ^15^ ^]^ Based on this hypothesis, we describe how a viral mimicry‐primed state can be induced by targeting the safeguards against aberrant NA including the epigenetic control, the DNA damage response, mitochondrial function, mRNA splicing, RNA editors, and exoribonucleases.^[^ ^72^ ^]^

### Epigenetic control

Initially, epigenetic therapies were found to be major drivers of retrotransposon reawakening.^[^ ^102^ ^]^ This is mainly because epigenetic control mechanisms target endogenous retroviral elements to establish repressive heterochromatin.^[^ ^103^ ^]^ Thus, it is not surprising that cancer‐associated mutations such as in H3.3,^[^ ^104^ ^]^ SMARCB1^[^ ^105^ ^]^ and DNMTA3A ^[^ ^106^ ^]^ can perturb the silencing of EREs and prime cancer cells for viral mimicry. Recently, it was identified that targeting these control mechanisms with DNA methyltransferase inhibitors (DNMTi) or inhibitors of facultative or constitutive heterochromatin writers as well as erasers of euchromatic histone marks, have been identified as a potent means to induce a viral mimicry‐primed state.^[^ ^107^, ^108^ ^]^ In addition, epigenetic therapy‐induced ERE transcription can also contribute to genome instability by increasing replication stress, mitotic errors and mutagenic transposition.^[^ ^109^ ^]^ However, to date, no study has investigated whether PARP7i may synergize with inhibitors of epigenetic control mechanisms.

### Genome integrity

Genomic instability, an innate feature of almost all cancer cells, can promote a type I IFN response by accumulating cytosolic MN and chromatin fragments or by derepressing retroviral elements.^[^ ^110^ ^]^ Thus, a second strategy to induce a viral mimicry‐primed state, is to target key components of the DNA damage response or introduce exogenous genotoxic stress.^[^ ^111^, ^112^ ^]^ Indeed, PARP1 inhibitors (PARPi), a class of PARP1‐selective small molecules, and radiotherapy have been found to promote type I IFN‐type signaling by increasing levels of aberrant NA.^[^ ^110^, ^113^ ^]^ In addition, a recent report described that the pan‐PARP inhibitor thioparib showed anti‐tumor activity. This was achieved by inhibiting PARP1, PARP2, and PARP7, which resulted in the accumulation of cytosolic DNA in a PARP1‐dependent manner. In addition, PARP7 inhibition induced type I IFN signaling.^[^ ^114^ ^]^ These findings suggest that the combination of PARPi or radiotherapy together with PARP7i may aid in tumor control. Although this review focuses primarily on ADP‐ribosylation, itis important to recognize that other druggable components of the DNA damage response, such as ATM,^[^ ^115^ ^]^ ATR^[^ ^116^ ^]^ and CHK1,^[^ ^117^ ^]^ have also been shown to have immunogenic effects when inhibited.

### Mitochondrial integrity

A third approach to trigger viral mimicry could be to stimulate the release of mtDNA and mtRNA by promoting mitochondrial dysfunction. Recently, fumarate has been shown to play a role in mtDNA‐ and mtRNA‐dependent innate immune responses. Pharmacological or genetic inhibition of fumarate hydratase, which converts fumarate to malate, increased the intracellular levels of fumarate and, in turn, activated the RLR‐MAVS and cGAS‐STING pathway.^[^ ^47^, ^50^ ^]^ Moreover, the mitochondrial membrane integrity was reduced or lost during sub‐lethal genotoxic stress, linking DNA damage‐induced type I IFN to the release of gDNA and mtDNA/mtRNA.^[^ ^46^ ^]^ Thus, mitochondrial health may co‐regulate the efficacy of PARP7i in inducing apoptosis and anti‐tumor immunity.

### mRNA splicing

A fourth potential approach is to target mRNA splicing components, as aberrant splicing is increased in cancer cells compared to healthy tissue.^[^ ^73^, ^75^ ^]^ The source of aberrant splicing can be found in spliceosome and non‐spliceosome associated mutations that dysregulate mRNA splicing and prime cancer cells for viral mimicry.^[^ ^118^ ^]^ These observations have encouraged the development of spliceosome inhibitors. Spliceosome‐targeted therapies do not simply stop splicing but rather modulate splicing through alternative branch point usage, which can increase the rate of aberrant RNAs, including intron retention events.^[^ ^119^ ^]^ mRNAs with abnormally retained introns have been described to form dsRNA structures and a large proportion of ERE in the genome are located in introns.^[^ ^120^ ^]^ Consistently, spliceosome‐targeted therapies have been found to increase cytosolic dsRNA levels, triggering a type I IFN response and CASP8‐mediated apoptosis.^[^ ^73^, ^75^ ^]^ Based on the similarities between the mode of cell death following PARP7 and spliceosome inhibition, it is tempting to speculate that spliceosome‐targeted therapies may enhance the anti‐tumor effect of PARP7i in FRA1‐driven cancer cells.

### RNA editors and nucleases

While all of the aforementioned studies describe mechanisms that would further increase the levels of self‐DNA and ‐RNA, alternative approaches attempt to enhance the immunostimulatory potential of already present NA.^[^ ^121^ ^]^ These approaches focus primarily on ADAR1, as it has been shown to be a vulnerability in cancer cells with elevated ISG scores.^[^ ^33^ ^]^ Furthermore, ADAR1 was found to modulate the response to PARP7i in a whole genome‐CRISPRi/CRISPRa screen.^[^ ^16^ ^]^ However, to date, there are no specific small molecule inhibitors targeting ADAR1.^[^ ^122^ ^]^ Therefore, a complete ADAR1 targeting strategy that includes the inhibition of ADAR1's deaminase activity and the Z‐RNA binding capacity of its p150 isoform is required to exploit the genetic dependence of ISG‐positive cancers on ADAR1 as well as to potentiate the effect of PARP7i.^[^ ^123^ ^]^ In addition to ADAR1, targeting m6A writers and erasers may provide an opportunity for a synergistic combination with PARP7i.^[^ ^124^ ^]^ Lastly, the exoribonuclease XRN1 has recently been identified as a repressor of dsRNA‐induced cell death, highlighting that cancer cells have evolved multiple strategies to evade an NA‐dependent innate response.^[^ ^35^, ^36^, ^125^ ^]^ Similar to ADAR1, efforts to target XRN1 using small molecules are only just emerging.^[^ ^125^, ^126^ ^]^

## CONCLUDING REMARKS

This review highlights the central role of PARP7 in regulating the cellular response to aberrant NA and its potential as a therapeutic target to induce viral mimicry. While PARP7i have shown promise in clinical trials,^[^ ^32^ ^]^ limitations such as off‐target effects and the risk of resistance remain. To overcome these challenges, the development of more selective PARP7i that minimize off‐target interactions is crucial. Furthermore, a deeper understanding of the mechanisms governing NA levels and their interplay with PARP7 is essential for the development of effective therapeutic strategies that exploit this pathway.

This review focuses on the concept of viral mimicry in FRA1‐driven and PARP7i‐sensitive cancers, where cells have elevated ISG scores due to the presence of aberrant NA. These cancer cells may be particularly susceptible to PARP7 inhibition, providing a unique therapeutic window. In addition, understanding NA‐sensing pathways in cancer has broader implications beyond PARP7i. It sheds light on fundamental mechanisms of innate immunity and immune evasion and may lead to the development of novel immunotherapies that target different aspects of this complex interplay.

Future research should focus on several key areas. First, to explore the efficacy of combining PARP7i with the therapeutic strategies outlined here, particularly in tumors with high ISG scores. Preclinical models can be used to evaluate the efficacy and safety of these combinations. Second, to elucidate the role of NA‐sensing pathways in the tumor microenvironment and how they influence immune cell function. Finally, to translate these findings into the clinical setting by designing and conducting clinical trials to evaluate the efficacy of these novel therapeutic approaches in cancer patients.

By addressing these areas, future studies can unlock the full potential of targeting NA‐sensing pathways and PARP7 for effective cancer immunotherapy. This will ultimately lead to improved patient outcomes and potentially revolutionize the treatment of cancer.

## CONFLICT OF INTEREST STATEMENT

The authors declare no competing interests.

## Data Availability

The data that support the findings of this study are openly available in the expression and dependency (CERES scores) datasets of the Dependency Map (DepMap) portal (https://depmap.org/portal/, release: Public 24Q2).
